# Clinical inertia and treatment intensification among patients with type ii diabetes mellitus at Debre Tabor comprehensive specialized hospital, Ethiopia: an institutional-based cross-sectional study

**DOI:** 10.3389/fendo.2025.1450928

**Published:** 2025-02-06

**Authors:** Samuel Berihun Dagnew, Samuel Agegnew Wondm, Getachew Yitayew Tarekegn, Abebe Tarekegn Kassaw, Tilaye Arega Moges

**Affiliations:** ^1^ Department of Clinical Pharmacy, College Health Sciences, Debre Tabor University, Debre Tabor, Ethiopia; ^2^ Department of Pharmacy, College of Health Sciences, Debre Markos University, Debre Markos, Ethiopia; ^3^ Department of Pharmacy, College of Health Sciences, Woldia University, Woldia, Ethiopia

**Keywords:** clinical inertia, treatment intensification, type 2 diabetes, Debre Tabor comprehensive specialized hospital, Ethiopia

## Abstract

**Background:**

People with type 2 diabetes mellitus who have clinical inertia often struggle to control their blood sugar levels and do not receive timely treatment intensification. Strict glycemic control has advantages, but many patients with diabetes are unable to reach their target blood sugar levels. The study’s main objective was to determine the prevalence of clinical inertia in patients with type 2 diabetes at Debre Tabor Comprehensive Specialized Hospital(DTCSH) in Ethiopia.

**Methods:**

An institutional based, cross-sectional research design was used at Debre Tabor Comprehensive Specialized Hospital from November 20/2023 to January 30/2024. A structured questionnaire modified from various medical records and literatures were used to gather data. A logistic regression model was also employed after the Hosmer-Lemeshow goodness-of-fit test was checked to find contributing variables to clinical inertia. A threshold of p < 0.05 was considered statistically significant.

**Result:**

In total, 287 samples were included in the research. The occurrences of clinical inertia 31.4% (95%CI: 25.9 - 36.8) were obtained from 90 patients. Aged patients (AOR = 1.103; 95% CI, 1.034 - 1.176; P = 0.003), medication fee (AOR = 4.955; 95% CI, 1.284 - 14.127; P = 0.020), medication nonadherence (AOR = 4.345; 95% CI, 2.457 - 15.537; P = 0.001), increase number of medication (AOR = 4.205; 95% CI, 2.657- 6.655; P ≤ 0.001), poor glycemic control (AOR = 2.253; 95% CI, 1.673 - 3.033; P ≤ 0.001) were more likely to have clinical inertia.

**Conclusion:**

One-third of patients experienced clinical inertia. Age, glycemic control, medication non-adherence, treatment fee, and number of medications were found to be strongly correlated with clinical inertia. More precise knowledge of the clinical inertia and the associated therapies is necessary to tackle this issue more effectively.

## Introduction

Diabetes is a widespread, problematical, progressive chronic illness that is the most urgent worldwide health issue, with an estimated 537 million individuals living with the disease in 2021 and an expected 783 million by 2045 (1). Critical, potentially lethal consequences of untreated diabetes include diabetic ketoacidosis (DKA) and hyperosmolar hyperglycemic state (HHS), both of which require direct medical attention. Over time, chronic hyperglycemia progressively harms vital organs. Blindness, end-stage renal disease, and amputation can have microvascular consequences, which include diabetes retinopathy, nephropathy, and neuropathy (2). The main causes of morbidity and mortality in patient with diabetes are macrovascular problems, such as peripheral artery disease, stroke, and cardiovascular diseases (CVDs) (3). Additionally, people with diabetes are disproportionately found in low- and middle-income nations, where access to treatment and a lack of infrastructure make the implications of untreated diabetes worse (4).

Clinical inertia is the primary and frequent issue with treating chronic diseases, particularly diabetes mellitus, and affects 463 million people globally including more than 30 million adults in the US (United States) (5, 6). According to the definition of clinical inertia, it is the inability to start or increase therapy as directed. “Clinical inertia” or “therapeutic inertia” is the term used to describe the discrepancy in diabetes management between recommendations and clinical practice. A patient experiencing clinical inertia does not meet evidence-based care goals because of a lack of therapy intensification (7–9). Clinical practice recommendations advocate gradual treatment intensification (TI) until the glycemic target is reached, along with frequent monitoring of hemoglobin A1c (HbA1c). Glycemic management, however, was frequently found to be insufficient (10, 11).

Diabetes-related clinical inertia lowers life expectancy, increases the likelihood of complications from the disease, and causes extended episodes of uncontrolled hyperglycemia (12–14). Keeping the glycemic level within the suggested ranges is advantageous to minimize diabetic-related complications (15). According to a study conducted on a large cohort of type 2 diabetic mellitus (T2DM), patients followed for 22 years, patients who delayed treatment intensification by 1 year had a considerably higher cause of myocardial infarction, heart failure, stroke, and a composite of cardiovascular events (16). In 2012, it was anticipated that 3.7 million people worldwide would die from high blood glucose, and between 2011 and 2030, the financial cost of diabetes is expected to reach US$1.7 trillion (17).

Factors that affecting the presence of clinical inertia how it affects patient care and the ensuing clinical ramifications in type 2 DM. The barriers that cause clinical inertia and how education might lessen its negative effects on patient care and treatment outcomes (18–20). Clinical inertia is typically complex and depends on factors specific to the patient, provider, and the entire system. It is estimated that around 30% of clinical inertia is caused by patient-related factors (21). A common occurrence that is particularly noticeable in patients with type 2 diabetes, but can affect the care of any medical condition, is the failure to start or escalate treatment or to follow evidence-based guidelines while taking therapeutic measures (22, 23). Taking action should reduce therapeutic and clinical inertia and result in better patient treatment outcomes (24).

The burden of diabetes has increased, and the prevalence of comorbidities is much higher among T2DM patients in Ethiopia. The processes that have been attempted to lower therapeutic inertia are critical to improving type 2 diabetes evidence-based therapy (5). The quality of treatment for people with diabetes remains below ideal levels, despite several therapeutic interventions that have been developed in the last ten years that concentrate on health system-level improvements in diabetes care (25). Consequently, it is essential to research clinical inertia in nations like Ethiopia, which struggle with issues including inadequate healthcare resources, a subpar healthcare system, low health literacy, and practitioners with inadequate training. This study aimed to assess clinical inertia on treatment intensification among patients with type 2 diabetes mellitus at Debre Tabor Comprehensive Specialized Hospital, in Ethiopia.

## Methods and materials

### Study Setting, design, and period

The study was carried out at Debre Tabor Comprehensive Specialized Hospital (DTCSH) between November 2023 to January 3024. Debre Tabor Comprehensive Specialized Hospital was established in Debre Tabor Town in 1923 E.C. Located 102 kilometers from Bahir Dar, the capital of Amhara Regional State, and 667 kilometers from Addis Ababa, Debre Tabor is the capital of the South Gondar Zone. In its catchment area, the hospital provides care approximately 3.5 million people. The hospital offers diabetic patient follow-up care in its chronic outpatient department (26, 27). A cross-sectional study design was used to assess clinical inertia at DTCSH.

### Study population and eligibility criteria

This study included all patients older than 30 years with T2DM who had follow-up appointments at Debre Tabor Comprehensive Specialized Hospital during the data collection period. Individuals excluded from the study of patients with type 2 diabetes mellitus owing to serious sickness, physical deformity, and breakfast consumption were based on metabolic syndrome and lifestyle factors. Patients diagnosed with type I diabetes or gestational diabetes within the indexing period, also were not eligible to participate in the study. Additionally, study participants with incomplete medical information were not included.

### Sampling size determination and sampling technique

Since no relevant previous study had been carried out in the study setting or other areas with similar population backgrounds, the sample size was determined using a single population proportion formula: n = Z2 p (1-p)/W, where n = sample size required, W = marginal error of 5% (w = 0.05), Z = the degree of accuracy required (95% level of significance = 1.96), and P = the proportion of clinical inertia in patients with T2DM treated, assumed to be 0.5(50%). n = 1.962 0.5(1-0.5)/0.052 = 384.16 = ~384.

The final sample size (NF) was determined using a correction procedure because the research population is fewer than 10,000. If NF=n/1+n/N, then 384/1 + 384/820 = 261.

Here, N is the overall study population and NF is the final sample size.

A 10% contingency was considered, and 287 study participants were enrolled. The study participants were selected using consecutive sampling.

### Operational definitions

#### Clinical inertia

Clinical inertia is identified when the HbA1c level was found at ≥ 7%, at the index date, followed by no treatment intensification from the index date and the subsequent prescription (28).

Treatment intensification: the addition of a new antidiabetic medication, switching from oral antidiabetic drugs (OAD) to an injectable medication, or raising the dosage of an already-existing antidiabetic medication without stopping or lowering the dosage of other antidiabetic medications (29).

Good glycemic control: HbA1c levels of less than 7% a; this is considered good glycemic control for most adults with diabetes (30).

Poor glycemic control: an average glycated hemoglobin (HbA1c) level of 7% or higher (31).

Adequate medication adherence: when a patient takes their medications as prescribed (32).

Inadequate medication adherence, also known as medication nonadherence, is when a patient doesn’t take their medication as prescribed (32).

Adequate exercise adherence: is when a person’s behavior follow their exercise plan (33).

Inadequate exercise adherence: is when someone doesn’t follow their exercise plan as closely as they should (33).

Adequate dietary adherence: is when a person follows the diet and lifestyle recommendations given to them by a healthcare provider (34).

Inadequate dietary adherence: is when someone doesn’t follow the recommended diet (35).

### Data collection tools, procedures, and measurement

After exploring several literatures (18, 28, 36–38), the data extraction tools were formed, with adjustments made based on the context and kind of patient medical information. Using various literature sources, the tool was structured to allow for the proper evaluations of clinical inertia and its contributing factors. The questionnaire was first created in English, then translated into Amaharic, the native tongue, and then back-translated into English to guarantee meaning consistency. The questionnaire’s Amaharic language version’s internal reliability (Cronbach’s α) was 0.82, suggesting good reliability. The questionnaire was divided into three sections: sociodemographic, clinical and laboratory, clinical inertia and therapeutic intensification. Following instructions regarding the study’s objectives, data collection tools and producers, and ethical considerations, two clinical pharmacists collected the data. Direct patient interviews were used to gather primary data, and patient medical records were used to documented laboratory results, medical conditions, and prescription dosages. By American Diabetes Association (ADA) guidelines, treatment adjustments, titrations, and intensifications were made (39).

### Outcome measurements

Outcomes were measured by using HbA1c The index date which is defined as the date of the first HbA1c laboratory test above the target level (HbA1c ≥ 7.00%) during the study. When an HbA1c level was ≥ 7.00% on the index date and there was no treatment intensification from that date and the subsequent prescription, clinical inertia was recognized. Using the two approaches, no clinical inertia was found. Initially, patients who had an HbA1c level of 7.00% or higher on the index date were given more intensive medication either on the index date or at a later prescription. Second, patients’ blood sugar levels were within the desired range, and they received treatment intensification at the subsequent follow-up time rather than at the index. Treatment intensification was evaluated one of the three ways; the addition of a new antidiabetic medication, switching from an OAD to an injectable medication, or raising the dosage of an already-approved medication without stopping or lowering the dosage of other antidiabetic medications (28, 39, 40).

### Data quality control

Before the actual data collection, the supervisor and data collectors received training about the objectives, data collection tools and processes, and ethical considerations. 10% of the sample size was used for the pretest, and certain changes were made. Before analysis, the supervisor and data collectors made sure the data was accurate and included all necessary information at each stage. If the management of medicine changed, doctors were notified. The investigator also specifically followed the protocols for gathering data. They closely observed alterations in medication experiences and inconsistent test results to detect any possible signs of clinical inertia.

### Data management and analysis

Following collection, the data were entered into Epidata version 4.6, cleansed, and STATA version 17 was used for analysis. The results of the descriptive statistics were summarized using tables and figures. A Q-Q plot and a histogram were used to look at the data’s normal distribution. Depending on how the data were distributed, continuous variables were presented using the mean (standard deviation) and median (interquartile range), whereas categorical variables were presented using frequency and percent.

A logistic regression model was used after checking of Hosmer-Lemeshow goodness-of-fit test. Therefore, the factors associated with clinical inertia were evaluated using the binary logistic regression model. Variables included in the multivariable logistic regression analysis have a P-value of less than 0.25 in the bivariable analysis. Ultimately, the 95% confidence interval (CI) for the adjusted odds ratio (AOR) was provided, and a P-value of less than 0.05 indicated statistical significance.

## Results

### Socio-demographic information

In this study, 287 people were involved. With a mean age of 55.19 ± 12.35, men were about half of the participants (50.52%). The majority (58.89%) of the study individuals were rural residents. Approximately two-thirds (71.08%) of the individuals adhered to their drug regimens adequately. On the other hand, the majority (80.49%) received their medication through payment (**Table 1**).

**Table 1:** Socio-demographic characteristics among patients with Type II diabetes mellitus at Debre Tabor Comprehensive Specialized Hospital, Ethiopia (N= 287).

Variables	Categories	Frequency (n)	Percentage (%)	Mean (± SD)
Age	31- 64	199	69.34	55.19 ± 12.35
	≥65	88	30.66	
Gender	Male	145	50.52	
	Female	142	49.48	
Marital status	Single	23	8.01	
	Married	189	65.85	
	Separated	70	24.40	
	Windowed	5	1.74	
Educational level	Unable to read & write	120	41.81	
	Primary	25	8.71	
	Secondary	92	32.06	
	College and above	50	17.42	
Religion	Orthodox	237	82.58	
	Muslim	50	17.42	
Place of residence	Rural	169	58.89	
	Urban	118	41.11	
Occupation	Employed	201	70.03	
	Unemployed	86	29.97	
Sources of drug	Payment	231	80.49	
	Free	56	19.51	
Alcohol	Yes	118	41.11	
	No	169	58.89	
Cigarette	Yes	16	5.57	
	No	271	94.43	
Medication adherence	Inadequate	83	28.92	
	Adequate	204	71.08	
Diet adherence	Adequate	67	23.34	
	Inadequate	220	76.66	
Exercise adherence	Adequate	261	90.94	
	Inadequate	26	9.06	

### Laboratory profile of study participants

The laboratory results that showed the greatest deviation were those of FBS and SGPT, with mean (± SD) values of 136.79 ± 55.48 and 46.00 ± 29.09, respectively, followed by HDL, at 39.97 ± 11.09 (**Table 2**).

**Table 2:** Laboratory profile among patients with type II diabetes mellitus at Debre Tabor Comprehensive Specialized Hospital, Ethiopia (N= 287).

Variables	Normal n (%)	Abnormal n (%)	Normal references range	Mean (± SD)
LDL	62 (21.6)	44 (15.3)	< 130 mg/dL	118.43 ± 34.84
HDL	45 (15.7)	38 (13.2)	> 40 mg/dL	39.97 ± 11.09
TC	48 (16.7)	32 (11.2)	< 200 mg/dL	175.57 ± 33.17
TG	35 (12.2)	24 (8.4)	< 150 mg/dL	142.12 ± 35.02
Serum creatinine	191 (68.6)	73 (25.4)	0.6 – 1.3 mg/dL	0.84 ± 0.27
Blood urea nitrogen	216 (75.3)	48 (16.7)	6 – 24 mg/dL	13.08 ± 4.93
SGPT	32 (11.2)	30 (10.5)	7 – 56 U/L	46.00 ± 29.09
SGOT	37 (12.9)	22 (7.7)	10 – 45 IU/L	33.59 ± 16.98
Fasting blood sugar	162 (56.4)	125 (43.6)	100 – 125 mg/dL	136.79 ± 55.48
HbA1C	163 (56.8)	124 (43.2)	<7%	7.28 ± 1.77
White blood cell	155 (54.0)	28 (9.8)	4.5 – 11.0 × 109/L	7.23 ± 2.92
Neutrophil	152 (53.0)	31 (10.8)	2.5 – 7.0*103/L	5.34 ± 3.57
Platelet	156 (54.4)	27 (9.4)	150 – 450*103/L	253.61 ± 90.04

HDL, high density lipoprotein; LDL, low density lipoprotein; TC, Total Cholesterol; TG, triglyceride; SGPT, serum glutamic pyruvic transferase; SGOT, glutamic-oxaloacetic transaminase; GbA1C, Hemoglobin A1C.

### Clinical pattern of study participants

The majority of study participants (81.53%) had been diagnosed with diabetes for fewer than 15 years, and the mean number of follow-up visits per year was 5.28 ± 2.37. About one-fourth (22.3%) of the patients experienced diabetic-related complications. The patients’ average number of medications and Charles comorbidity index were 2.31 ± 1.47 and 2.06 ± 1.61, respectively (**Table 3**).

**Table 3:** Clinical pattern of study participants among patients with type II diabetes Mellitus at Debre Tabor Comprehensive Specialized Hospital, Ethiopia (N= 287).

Variables	Categories	Frequency (n)	Percentage (%)	Mean (± SD) or Median (IQR)
Duration of diabetes mellitus (year)	<15	234	81.53	6 (3-11) years
	≥15	53	18.47	
Number of follow-up per year	<4	67	23.34	5.28 ± 2.37 days
	≥4	220	76.66	
Comorbidity	No	68	23.69	
	Hypertension	78	27.18	
	Infections	74	25.78	
	Dyslipidemia	69	24.04	
	Heart failure	46	16.03	
	Coronary artery disease	38	13.24	
	Respiratory (Athema & COPD)	31	10.80	
	Rheumatoid disease	27	9.41	
	Neurology disorder	22	7.67	
	Thyroid disorder	6	2.09	
Complications	Microvascular	35	12.19	
	Macrovascular	29	10.11	
Class of Medications	Metformin + Sulfonylureas	145	50.52	
	Metformin + Insulins	96	33.45	
	Metformin	41	14.29	
	Metformin + DPP4	5	1.74	
Charlson comorbidity index				2.06 ± 1.61
Number of Medications				2.31 ± 1.47
Types of Doctors	General practitioners	130	45.30	
	Specialists	157	54.70	
Non-Pharmacologic interventions	No	79	27.53	
	Yes	208	72.47	

NB, COPD, Chronic Obstructive Pulmonary Disease; DPP4, Dipeptidyl Peptidase-4 Inhibitors; SD, Standard Deviation; SD, Standard Deviation; IQR, Inter Quartile Range.

### The development of clinical inertia

**Figure 1:**
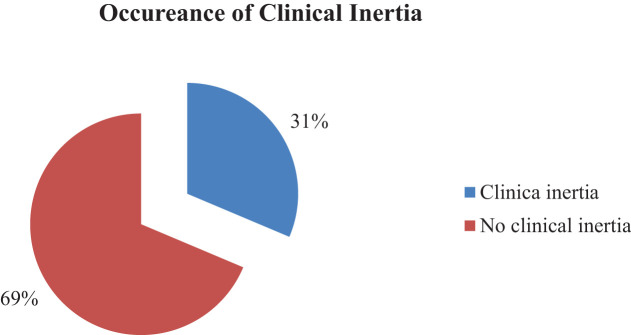
The occurrence of clinical inertia among type 2 diabetes in Debre Tabor Comprehensive Specialized Hospital, Ethiopia.

Clinical inertia in patients with type 2 diabetes was assessed in this study. Accordingly, one-third 31.4% (95% CI: 25.9 - 36.8) of the patients had clinical inertia, while the remaining patients did not (**Figure 1**).

### Clinical pharmacist intervention on clinical inertia

**Figure 2:**
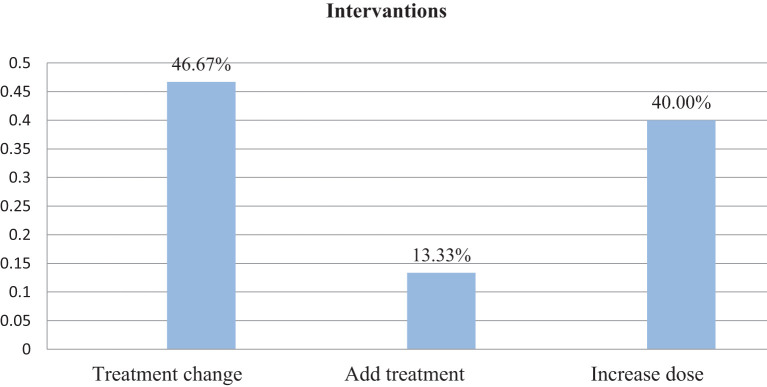
Intervention given following the onset of clinical inertia among patients with type 2 diabetes at Debre Tabor Comprehensive Specialized Hospital, Ethiopia.

Within this investigation, clinical inertia was present in 31% of participants. Aiming to overcome clinical inertia, clinical pharmacists took part. Thus, of the total interventions offered, 46.67% were changing the course of therapy, 40.00% were raising the dosage, and 13.33 involved were new course of action (**Figure 2**).

### Factors that affect the presence of clinical inertia

Using a binary logistic regression model, the determinate variables of clinical inertia occurrences were found. As a result, multivariable binary logistic regression analysis showed that clinical inertia was significantly associated with age, glycemic control, medication nonadherence, sources of medical care, and quantity of medications.

Additionally, if all other factors stay the same, the development of clinical inertia will happen 1.1 times more frequently as the patient becomes older (AOR = 1.103; 95% CI, 1.034 - 1.176; P = 0.003). Compared to patients receiving their medication for free, those receiving payment for their treatment experienced 5 times (AOR = 4.955; 95% CI, 1.284 - 14.127; P = 0.020) higher levels of clinical inertia. Patients with inadequate medication adherence had clinical inertia 4.3 times more frequently than those with adequate medication adherence (AOR = 4.345; 95% CI, 2.457 - 15.537; P = 0.001). As the number of Medications increased the occurrence of clinical inertia was times 4.2 (AOR = 4.205; 95% CI, 2.657- 6.655; P ≤ 0.001). Compared to individuals with adequate glycemic control, those with poor glycemic control had 2.3 times greater clinical inertia (AOR = 2.253; 95% CI, 1.673 - 3.033; P ≤ 0.001) (**Table 4**).

**Table 4:** Factors that affect the presence of clinical inertia among patients with type II diabetes mellitus at Debre Tabor Comprehensive Specialized Hospital, Ethiopia (N= 287).

Variables	Category	Inertia		COR CI 95%	P-Value	AOR CI 95%	P-Value
		Yes (90)	No (197)				
Age	55.19 ± 12.35	64.27 ± 10.62	51.05 ± 10.78	1.107 (1.078 - 1.136)	≤0.001	1.103 (1.034 - 1.176)	**0.003**
BMI	<25	66	178	1		1	
	≥25	24	19	3.407 (1.752 - 6.623)	≤0.001	2.076 (0.455 - 9.463)	0.345
Sources of Medicine	Free	7	49	1		1	
	Payment	83	148	3.926 (1.701 - 9.059)	0.001	4.955 (1.284 - 14.127)	**0.020**
Exercise adherence	Adequate	78	183	1		1	
	Inadequate	12	14	2.011 (0.889 - 4.545)	0.093	3.673 (0.702 - 13.236)	0.124
Diet Adherence	Adequate	76	144	1		1	
	Inadequate	14	53	1.998 (1.042 - 3.832)	0.037	0.434 (0.112 - 1.682)	0.227
Medication Adherence	Adequate	70	134	1		1	
	Inadequate	20	63	1.646 (0.921- 2.939)	0.092	4.345 (2.457 - 15.537)	**0.001**
Duration DM	<15	62	172	1		1	
	≥15	28	25	3.107 (1.684 - 5.732)	≤0.001	1.392 (0.393 - 4.938)	0.608
Number of Medication	2.31 ± 1.47	3.44 ± 1.53	1.29 ± 0.79	3.563 (2.691- 4.718)	≤0.001	4.205 (2.657- 6.655)	**≤0.001**
Glycemic Control (HbA1c)	7.28 ± 1.77	8.65 ± 1.63	6.65 ± 1.45	2.151 (1.756 - 2.634)	≤0.001	2.253 (1.673 - 3.033)	**≤0.001**
Complication	Yes	25	26	2.529 (1.362 - 4.697)	0.003	0.457 (0.106 - 1.968)	0.293
	No	65	171	1		1	
Comorbidity	Yes	30	38	2.092 (1.191 - 3.675)	0.010	0.525 (0.131 - 2.098)	0.362
	No	60	159	1		1	
CCI	2.06 ± 1.61	3.06 ± 1.66	1.59 ± 1.37	1.828 (1.516 - 2.206)	≤0.001	0.897 (0.518 - 1.554)	0.699
Types of Doctor	GP	53	77	2.232 (1.343 - 3.711)	0.002	1.153 (0.425 - 3.128)	0.779
	Specialist	37	120	1		1	

NB: AOR, Adjusted odds ratio; COR, crude odds ratio; CI, confidence interval; bold indicated p value < 0.05, CCI, Charlson comorbidity index; BMI, body mass index; HA1C, hemoglobin A1C.

## Discussion

Diabetes is a severe, chronic illness that is very common and associated with increased mortality, morbidity, and medical expenses (41). Poor glycemic control is a major problem in the treatment of people with type 2 diabetes. Prolonged poor glucose control can be caused by clinical inertia. The main cause of poor treatment outcomes in the management of type 2 diabetes is the primary care practitioner’s “recognition of the problem but failure to act” is clinical inertia (24, 42). This study aimed to determine the prevalence of clinical inertia among type two diabetes patients at the Debre Tabor Comprehensive Specialized Hospital in Ethiopia.

According to this study, 31% of patients with T2DM had clinical inertia in actual clinical settings at Debre Tabor Comprehensive Specialized Hospital in Ethiopia. This incidence was lower than that of a prior study (43–45) which had a prevalence of clinical inertia from 49.9% -72.8%. However, higher than a study conducted in Thailand 26.2% (28), in another study in Thailand 24.0% (46), and in the United Kingdom 26.25% (47). In ordinary clinical practice, this data indicates a delay in treatment escalation with anti-diabetic medicine. The high prevalence of clinical inertia was due to inadequate time and resources to deal with patient concerns, overestimating the level of care given, poor communication between patients and healthcare providers, noncompliance with medication regimens, patient attitudes and beliefs, and challenges about the community and culture could have variable.

Various factors contribute to the complex nature of clinical inertia. In contrast to earlier research (37, 48), our finding revealed a greater chance of clinical inertia in older people than in adults. Despite being the ones who could benefit most from intensification with high-efficacy medications, they were less likely to obtain treatment intensification with injectable drugs. This is because of patients’ levels of understanding in many areas and physicians’ awareness of this is the reason. However, previous studies have shown that older patients were more likely than adult patients to experience clinical inertia, which is consistent with our findings (18, 28). Physicians tend to increase treatment intensity because elderly patients are more likely than younger patients to experience hypoglycemia. Furthermore, participants with fewer comorbidities were selected for this investigation. People with several comorbidities typically take many medications. Drug-drug interactions should be considered in cases of treatment intensification.

Without identifying out-of-pocket expenses as a deterrent to intensifying a patient’s drug regimen, clinical inertia is not conceivable (49). When it comes to clinical inertia, financial considerations are the primary barrier to selecting the best course of action for patients, including therapeutic optimization. We found that patients who paid for their medication had a 5-fold increased risk of developing clinical inertia compared to those who received it for free. This was proved by a study conducted in the United States (24, 50). The fact that it is difficult for these patients to afford and use medication may also be a sign of their stated mistrust of their ability to adhere to treatment regimens. In addition to the cost of the medication, the expenses of follow-up visits for medication titration and lab draws for assessing the safety and effectiveness of new medications should also be taken into account in addition to the medications.

Medication non-adherence remains a significant barrier to achieving optimal glycemic control in patients with type 2 diabetes (51). In our study, the likelihood of clinical inertia was four times higher in patients with inadequate medication adherence than in those with acceptable medication adherence. This finding is consistent with the American Diabetes Association indicating that better medication adherence may result in greater treatment intensification (52). Inadequate adherence to treatment regimens results in clinical inertia, preventable morbidity and mortality, and medical expenses and utilization (53). Indifferent to each other, clinical inertia and non-adherence lead to inadequate glycemic control.

According to our research, the a chance of developing clinical inertia quadruples as more drugs are used. which were corroborated by earlier research, showed that experiencing clinical inertia was linked to using more medications at the index date (28, 54, 55). Another study discovered that compared to individuals with fewer OADs, those with more OADs saw noticeably more insulin treatment intensification (56). Among polypharmacy patients, there was a more noticeable delay in therapy intensification. Contrary to other research, there is still uncertainty regarding the relationship between the quantity of medications used and clinical inertia in T2DM. Individuals who were already on a lot of medications were more expected to experience clinical inertia since adding more antidiabetics is probably not what these patients would like to hear. Furthermore, polypharmacy, medication-related adverse events, and drug-drug interactions increased with treatment intensification.

As per our findings, there is a two-fold greater correlation between poor glycemic control and clinical inertia compared to adequate glycemic control, which a prior study has supported (57, 58). Poor glycemic control makes it obvious that type 2 diabetics should start receiving more intense therapy early. The amount of time a patient’s HbA1c level was above a threshold over a given period is known as their “glycemic burden.” Various studies varied greatly in their thresholds for all other readings and in how long they took to evaluate clinical inertia (59). Furthermore, a huge number of previously treated patients remain to have poor glycemic control and do not obtain prompt and appropriate therapy intensification. Physician adherence to recommended practices may be aided by increased knowledge of glycemic control targets, A1c result notification systems, and resources for executing aggressive glucose lowering.

### Strengths and limitations of the study

Because this is the initial research of its kind in Ethiopia, the results will provide a starting point for other researchers who want to further investigate further. This study used a cross-sectional observational design, which made it impossible to examine how the intervention worked out. One of the study’s limitations is that it is a single center, so the results could not be generalized. We also didn’t know why doctors hadn’t added new medications or raised the dosages of existing ones. Many aspects of community hospitals and lower-level hospitals would be engaged, including drug availability and physician knowledge. Although these findings are important, care should be taken when interpreting them.

## Conclusion

In conclusion, clinical inertia developed in one-third of patients. There was a strong correlation between clinical inertia and age, glycemic control, medication non-adherence, sources of medical treatment, and number of drugs. To overcome clinical inertia, efforts should be directed toward glycemic control, timely therapeutic changes, improving drug adherence through assistance and education, guaranteeing access to high-quality healthcare, and optimizing treatment regimens by reducing needless polypharmacy. Therefore, more precise knowledge of the clinical inertia and the associated therapies is necessary to tackle this issue more effectively.

## Data Availability

The original contributions presented in the study are included in the article/supplementary material. Further inquiries can be directed to the corresponding author/s.
